# THE ROLE OF FOXO3 TRANSCRIPTION FACTOR IN ALZHEIMER’S DISEASE PATHOLOGY

**DOI:** 10.1093/geroni/igz038.3102

**Published:** 2019-11-08

**Authors:** Shuqi Du, Laure Maneix, Qinghao Zhang, Ying-Wooi Wan, Hui Zheng

**Affiliations:** Baylor College of Medicine, Houston, Texas, United States

## Abstract

Alzheimer’s disease (AD) is a progressive and degenerative brain disease and age is one of its strongest risk factors. Aging is a complex process but it is reasonable that a delayed aging process may lower the risk of AD or postpone its pathogenesis. Studies in C. elegans revealed that the activity of DAF-16 is required for the life span extension in many long-lived strains. The mammalian homologs for DAF-16 are the Forkhead box O (FOXO) transcription factors. FOXO3, one of the members in the FOXO family, has been identified in several studies as a susceptibility gene for human longevity. I found that the expression level of Foxo3 in the mouse brain decreases with age or in an AD mouse model. To further study the role of Foxo3 in AD, I generated Foxo3 conditional knockout mice which depletes Foxo3 in neural cells. These mice have reactive astrogliosis in the cortex and also upregulation of some astrocytes specific markers like Gfap and Aqp4. In vitro culture of primary astrocytes from the knockout mice shows impaired respiratory capacity in the Seahorse mito stress test, which corresponds to the expression level change of metabolic genes like Acot1. When we bred the knockout mice with 5xFAD, a mouse model for amyloid pathology. I found increased plaque load and core plaque size in the cortex of the mice with Foxo3 deficiency. This study shows the critical function of FOXO3 in maintaining brain homeostasis and provide new insights into AD pathogenesis and drug discovery.

